# Seroprevalence of hepatitis E virus in risk populations and blood donors in a referral hospital in the south of Brazil

**DOI:** 10.1038/s41598-021-85365-5

**Published:** 2021-03-16

**Authors:** Marisa Boff Costa, Michele Soares Gomes Gouvêa, Samira Chuffi, Gustavo Hirata Dellavia, Felipe Ornel, Lísia Von Diemen, Félix Kessler, João Renato Rebello Pinho, Mário Reis Álvares-da-Silva

**Affiliations:** 1 Graduate Program in Gastroenterology and Hepatology, Universidade Federal do Rio Grande do Sul (UFRGS), Porto Alegre, Brazil; 2 Laboratório de Gastroenterologia e Hepatologia Tropical, Departamento de Gastroenterologia, LIM/07, Instituto de Medicina Tropical da USP, Hospital das Clinicas HCFMUSP, School of Medicine, University of São Paulo, São Paulo, Brazil; 3 School of Medicine, UFRGS, Porto Alegre, Brazil; 4 Centro de Pesquisa em Álcool e Drogas, Graduate Program in Psychiatry, School of Medicine, UFRGS, Porto Alegre, Brazil; 5 GI/Liver Unit, Hospital de Clínicas de Porto Alegre, Porto Alegre, Brazil

**Keywords:** Viral hepatitis, Liver cirrhosis, Viral hepatitis

## Abstract

The prevalence of anti-hepatitis E virus (HEV) antibodies has a high heterogeneity worldwide. South American data are still scarce. The aim of this study was to evaluate the prevalence of HEV in populations at risk in comparison to blood donors (BD). A cross-sectional study was carried out in adults of different risk populations including crack users (CK), residents in a low income area (LIA), cirrhotic (CIR) and liver transplant patients (LT) compared with BD. The WANTAI HEV ELISA test was used and real-time PCR (in-house for screening and ALTONA as confirmatory test) for HEV RNA screening. A total of 400 participants were included. Anti-HEV IgG was positive in 19.5% of the total sample, reaching the highest rate in the CIR group, 22.5%, followed by CK, LT, and LIA (20%, 18.7%, and 17.5%, respectively). The prevalence found in BD individuals was of 18.7% (p = NS). Anti-HEV IgM was positive in only 1.5% of the sample (6/400). No blood or stools samples were positive for HEV RNA. The seroprevalence reported is among the highest rates ever found in Brazil. Considering the intense diagnostic investigation, data show that HEV circulation is more common that might be expected in our country.

## Introduction

Hepatitis E, caused by hepatitis E virus (HEV), is one of the main causes of acute hepatitis in the world^1^. However, its epidemiological profile has demonstrated that the disease is not limited to developing countries and its clinical implications are beyond of just an acute infection^2,3^. The World Health Organization (WHO) estimates that hepatitis E caused approximately 44.000 deaths by 2015, accounting for 3.3% of viral hepatitis mortality^4^. It is known that HEV can cause chronic disease at-risk populations, especially in immunocompromised patients. Recent studies have revealed that HEV viremia in asymptomatic blood donors in Europe is more frequent than previously assumed^3,5^. In addition, approximately 0.04 to 0.12% of blood donations are detected by viral RNA^6^, demonstrating a higher prevalence than that identified in donors of blood for hepatitis B (HBV), hepatitis C (HCV) or immunodeficiency human (HIV) viruses. Anti-HEV seroprevalence in the US and Europe did not differ significantly (p = 0.25), while the rate in South America was significantly lower than that found in Europe (p = 0.04)^7,8^.

In Brazil, prevalence differs according to the region, population and the methodology used. The overall prevalence in Brazil seems to be lower if compared to European data. A recent meta-analysis demonstrated a 6% prevalence rate^9^. However, the prevalence in blood donors is around 10%^10,11^. In the last 5 years only two studies have found positive results for HEV viral load in Brazil, with a prevalence ranging from 0.2^12^ to 10%^13^. The aim of this study was to evaluate the prevalence of hepatitis E in populations at risk comparing their results to those obtained in blood donors.

## Results

Four hundred samples from five population groups were analyzed, four of them characterized as risk populations (CIR, LT, CK and LIA) and one from blood donors (BD), each group with 80 individuals. The mean age was 47.3 ± 15.8 years-old, 34.4% were female and 65.6% were male.

Regarding the epidemiology of the sample, Table 1 describes the findings of 320 individuals. A total of 110 women and 210 men were included. The seroprevalence of Anti-HEV IgG was higher in males (45/210 = 21.4%), however did not present a substantial difference in relation to females (19/110 = 17.2%). The mean age was 47.3 years, but the individuals in the CIR and LT groups presented a higher mean age compared to the other groups. Seroprevalence varied by age. The analysis of the presence of the individual HEV within the groups shows that the seroprevalence is higher as the age increases.

**Table 1:** Sociodemographic characteristics in different risk group for hepatitis E.

	Group				ELISA_HEV_IgG		Seroprevalence	
	CIR (n = 80)	LT (n = 80)	CK (n = 80)	BD (n = 80)	Positive (n = 49)	Negative (n = 189)	HEV IgG positive (%)	#
**Age**								
Average age	58 ± 10.7	59.5 ± 10.8	32.9 ± 7.8	38.6 ± 12	52.7 ± 13.5	49.4 ± 16.2	8.8	
18–39 year	4 (5.0)	5 (6.3)	65 (81.3)	44 (55.0)	19 (29.7)	99 (39.0)	16.1	a
40–59 year	40 (50.0)	32 (40.0)	15 (18.8)	30 (37.5)	25 (39.1)	91 (35.8)	21.3	a
≥ 60 year	36 (45.0)	43 (53.8)	0 (0)	6 (7.5)	20 (31.3)	64 (25.2)	23.5	a
**Sex**								
Female	35 (43.8)	36 (45)	0 (0)	39 (48.8)	19 (29.7)	9 (35.8)	17.3	a
Male	45 (56.3)	44 (55)	80 (100)	41 (51.3)	45 (70.3)	163 (64.2)	21.4	a
**Region of origin**								
Capital	42 (52.5)	36 (45)	58 (72.5)	38 (47.5)	34 (53.1)	139 (54.7)	19.5	a
Metropolitan	38 (47.5)	44 (55.0)	22 (27.5)	42 (52.5)	30 (46.9)	115 (45.3)	20.5	a
**Race**								
White	74 (92.5)	75 (93.8)	52 (65)	59 (73.8)	55 (85.9)	203 (79.9)	21.2	a
Black	3 (3.8)	4 (5)	14 (17.5)	11 (13.8)	2 (3.1)	30 (11.8)	6.3	b
Other	3 (3.8)	1 (1.3)	14 (17.5)	10 (12.5)	7 (10.9)	21 (8.3)	25.0	a
**Degree of education**								
None	1 (1.3)	2 (2.5)	0 (0)	ND	0 (0)	3 (1.6)	0	-
Elementary	51 (63.7)	43 (66.2)	50 (62.5)	ND	30 (61.2)	112 (59.2)	20.8	a
High	21 (26.2)	22 (27.53)	25 (31.2)	ND	12 (24.4)	56 (29.6)	17.6	a
Higher	7 (8.7)	13 (16.2)	5 (6.2)	ND	7 (14.2)	18 (9.5)	28.0	a

*CIR* cirrhotic patients, *LT* liver transplant patients, *CK* crack users, *LIA* residents in a low income area, *BD* blood donors, *ND* not available.

None: refer to individuals who did not complete primary school but are able to write and/or read.

Summary by frequencies (%).

Test for proportions difference, same letter across rows indicate not significant difference at 5%.

The predominant skin color in the sample was white (n = 260/320, 81.3%). This socio-demographic profile is common to all groups and applicable to anti-HEV IgG positive cases (n = 55/64; 85.9%). The black race had a lower seroprevalence (n = 2/64, 3.1%) with a statistically significant difference in relation to the others (p < 0.05). The educational level of the studied population was obtained from information of registry in the electronic medical record. For methodological reasons, this variable was not collected in the BD group. The degree of instruction corresponding to elementary education was present in 60% of the sample (n = 144/320), of these, 20.8% (30/144) presented Anti-HEV IgG positive. The higher education degree presented a higher seroprevalence of Anti-HEV positive (7/25 = 28%), without difference substantial in comparison to Anti-HEV negative.

Considering the geographic distribution, the city of Porto Alegre, capital of the State of Rio Grande do Sul, presented the highest prevalence (53.1%).

The anti-HEV IgG antibody was positive in 19.5% of the total sample, reaching the highest rate in the CIR group, 22.5%, followed by CK, LT, and LIA (20%, 18.7%, and 17.5%, respectively). The seroprevalence found in BD individuals was of 18.7% (p = NS). Two patients (2.5%) of the LT group and one (1.25%) of the BD group presented results compatible with limit of detection that characterized them as borderline, and the samples were retested and the borderline results were confirmed. The percentages are shown in Table 2. The anti-HEV IgM antibody was positive in only 1.5% of the sample (6/400), among these six positive samples, three were from the CK group (3.75%). Only one patient presented the isolated IgM fraction, the others presented simultaneous positivity for the IgM and IgG fractions.

**Table 2:** Seroloprevalence data of Anti-HEV. WANTAI HEV IgG and HEV IgM ELISA.

Seroprevalence	Overall	CIR	LT	CK	LIA	BD
Anti-HEV IgM	1.5% (6)	1.25% (1)	0% (0)	3.75% (3)	1.25% (1)	1.25% (1)
Anti-HEV IgG	19.5% (78)	22.5% (18)	18.7% (15)	20% (16)	17.5% (14)	18.7% (15)
Borderline	0.75% (3)	0% (0)	2.25% (2)	0% (0)	0% (0)	1.25% (1)

*CIR* cirrhotic patients, *LT* liver transplant patients, *CK* crack users, *LIA* residents in a low income area, *BD* blood donors, *BD* blood donors.

No plasma or stool samples were positive for HEV RNA by PCR. The negative PCR results obtained from the anti-HEV IgM samples were confirmed using the commercial kit (ALTONA—REALSTAR HEV RT-PCR, Hamburg, Germany).

## Discussion

The results demonstrate that HEV circulation is high in South Brazil, as confirmed by the overall seroprevalence. In Brazil, in the last 5 years, the prevalence rates found ranged from 0.3 to 22%. The results obtained from the MIKROGENS brand test vary from 0.3 to 15%, and results from the WANTAI brand test reach margins ranging from 9.8 to 22.5%. In two studies conducted in São Paulo, BR, analyzing HCV chronic patients and liver transplanted the authors found 12.0% and 8.1% respectively. In both studies MIKROGEN kit was applied^14,15^. The studies that found the highest prevalence of HEV in Brazil were performed with the WANTAI test, which is suggested to present higher sensitivity and specificity for HEV^16–18^. Three other Brazilian studies, performed in Southeast region, found a seroprevalence of 10.2 to 13.2%, evaluating patients with chronic HCV infection, using the WANTAI brand test^19,20^. Another study also performed ant-HEV with WANTAI test, evaluating cocaine and crack cocaine users in Midwest region, found a prevalence of 14.2%^21^. The results we found in this study were similar to those described in pregnant women and blood donors also in southern Brazil^22,23^ and higher than the previously described in other regions of Brazil even when the most sensitive methodology (WANTAI) was used. These results can be related to the intense European immigration to the region and the maintenance of traditional habits, such as high consumption of raw and undercooked pork meat. It is important to note that HEV is present in the swine in our country. Several studies are consistent with the presence of HEV, including HEV RNA, in swine herds in Brazil^24–27^. Studies conducted in Europe with the general population, patients with HIV, solid organ receptors, patients with chronic liver disease and individuals in contact with wild animals / pigs, show a prevalence ranging from 0.6 to 52.5%^7^.

Recent meta-analysis has evaluated the prevalence in blood donors (WANTAI-tested.) Data show that France, Poland and The Netherlands are the countries with the highest prevalence (52.5, 49.6 and 31%, respectively). New Zealand, Scotland, Australia, Canada and Ireland have a prevalence of less than 10%^28^. With other test—DIAGNOSTIC BIOPROBES (Genelabs diagnostics), blood donors from France presented a prevalence of 3.20%^29^, while in the same population, when tested with WANTAI kit the prevalence reached 22%^30^. In a cohort study, historical samples from the 1980s, 1990s, and 2000 to 2013, collected at two Danish hospitals, were prospectively analyzed using the WANTAI test. The prevalence found was 23.1%, 22.9% and 23.7%, respectively. No evidence of persistent HEV infection was found in this analysis. These data demonstrate that the virus has been circulating for decades in Europe with high prevalence but does not appear to cause disease in healthy populations^31^. All these findings are consistent with the results found in our study. In addition, as in Brazil, cases of active liver disease were not frequent. However, as the clinical implications of viral infection are unknown, groups at risk, especially those transplanted from solid organs, should be monitored for persistent hepatic changes.

The present study was made up of groups that have specific characteristics that condition them as a risky population, both for the contamination and for the establishment of chronic HEV infection. It is important to note that most patients remained asymptomatic and have mild but persistent liver abnormalities. In addition, some patients with immunological involvement have negative anti-HEV IgM or IgG serology, and in this sense, according to EASL 2018 guideline, it is mandatory that such patients be evaluated using nucleic acid amplification techniques (NATs) using serum or plasma and, if possible stool specimens^2^.

This single center study has limitations, including its retrospective analysis of three out of five groups, the scarce epidemiological data in some of these populations, and the relative small sample size.

A comprehensive diagnostic investigation, including full molecular analysis in serum samples, plasma, and stool of patients with cirrhotic and liver transplanted patients is one of the study’s strengths. In fact, in Brazil there is no other study that investigated the presence of the virus in the stool. The molecular test applied in this study was developed in-house, and presented sensitivity equivalent to the available commercial tests^32^. Samples that had evidence of amplification were subjected to two other amplification protocols: a conventional PCR selected as the most sensitive among many tested^33^ and a commercial PCR kit. Stool samples were also analyzed.

In conclusion, the results show the seroprevalence reported is among the highest rates ever found in Brazil and the HEV circulation is more common that might be expected in our country.

## Methods

A cross-sectional study was conducted in adults (≥ 18 years old) of both genders, with analysis of clinical and laboratory data from different populations at risk for HEV infection compared to control population constituted by blood donors (BD) from Southern Brazil. The population at risk included crack users (CK), residents of a low income area (LIA), cirrhotic (CIR) and liver transplant patients (LT). The samples were collected between 2013 and 2018. CK patients were recruited after emergency hospitalization at the Psychiatry Division of Hospital de Clínicas de Porto Alegre (HCPA). The participants of the LIA group were randomly recruited in individuals living in a popular neighborhood in the city of Porto Alegre. Patients in the CIR and LT groups were randomly selected from those in follow-up at the HCPA Liver Unit. Patients with cirrhosis of any etiology, confirmed by clinical, laboratory, radiological or pathological criteria, were included in the CIR group. Patients in the LT group were recruited among orthotopic liver transplant recipients under immunossupressive therapy. Blood donors are voluntary and healthy individuals, and all of them tested negative for hepatitis B and C, Chagas disease and syphilis. To ensure that there was no overlap between the populations studied, the recent blood donation was an exclusion criterion for groups CIR, LT and LIA. The CK users are not eligible for blood donation. To ensure that there was no overlap between the populations studied, the recent blood donation was an exclusion criterion for groups CIR, LT and LIA. The CK users are not eligible for blood donation. The demographic, epidemiological, clinical and laboratory data of the CK, CIR, LT and BD groups were obtained from electronic medical record information.

The ELISA technique was performed for the qualitative detection of IgM class antibodies and IgG (WANTAI, Beijing, China). All analysis were performed according to the manufacturer's instructions. The absorbance (A) was evaluated in a spectrophotometer (ZENYTH 200 rt) at 450 nm. The cut-off (C.O) was calculated for the evaluation of the results obtained (Negative A/C.O. ≤ 1; Positive A/C.O. ≥ 1; Borderline A/C.O. = 0.9–1.1).

In the CK, LIA and BD groups, the occurrence of active infection through the detection of viral RNA in the blood was investigated in patients with IgM and/or IgG positive samples. In the CIR and LT groups, the study was performed on blood and stool samples from all patients, regardless of the serology result. In order to perform the in-house PCR^32^ plasma samples (140 uL) and stool were initially subjected to RNA extraction using a QIAmp kit RNA Viral mini Kit (QIAGEN, Hilden, Germany). The stool samples were submitted to a preparation prior to the extraction using a protocol described by Leblanc et al.^34^.

For real-time PCR amplification was using the QuantiFast Pathogen RT-PCR + IC kit (QIAGEN) and TaqMan primers and probe that aligned in a highly conserved region (ORF3) of the HEV genome. The detection limit predicted for this PCR was 240 IU/mL (95% confidence interval: 173-513)^32^. Patient samples were tested in triplicate with negative controls included in each run. Positive samples were retested with a commercial kit—ALTONA REALSTAR, according to the manufacturer’s guidelines^34^. In an attempt to confirm the results, these samples were also subjected to a nested PCR reaction using primers that were aligned in MTase (Methyltransferase) region of ORF1 (HEV 1679/HEV 1680 and HEV 1681/HEV 1682 for the first and second steps of the nested PCR, respectively) producing a final product of 172 bp, which in previous studies showed better sensitivity and possibility of detection of different HEV genotypes^33^.

A convenience sample study was performed. It means that samples from CK, LIA and BD groups were already available at the HCPA from previously approved studies. All statistical analyzes were performed with the IBM SPSS program (SPSS Inc, Chicago, IL). Categorical variables were represented by absolute (n) and relative (%) frequencies and associations with positive and negative IgG were investigated by Fisher’s exact test for 2 × 2 crosses and by Chi-square test for > 2 × 2.

This study was approved by the Hospital de Clínicas de Porto Alegre ECR, and was registered on line (www.saude.gov.br/plataformabrasil: CAAE Ref. no. 68635817.0.0000.5327). The study was conducted according to the Strobe Guidelines and the Ethical Standards of the Declaration of Helsinki. The researchers signed a commitment to use the data, ensuring the anonymity and confidentiality of the information. All participants were volunteers, and all procedures for this study were started only after participants had read and signed an informed consent form approved by the HCPA Research Ethics Committee.

## References

[1] Colson P, Dhiver C, Gérolami R (2008). Hepatitis E virus as a newly identified cause of acute viral hepatitis during human immunodeficiency virus infection. Clin. Microbiol. Infect..

[2] European Association for the Study of the Liver (2018). EASL clinical practice guidelines on hepatitis E virus infection. J. Hepatol..

[3] Ahmed A, Ali IA, Ghazal H, Fazili J, Nusrat S (2015). Mystery of hepatitis e virus: Recent advances in its diagnosis and management. Int. J. Hepatol..

[4] Kamar N, Pischke S (2018). Acute and persistent hepatitis E virus genotype 3 and 4 Infection: Clinical features, pathogenesis, and treatment. Cold Spring Harb. Perspect. Med..

[5] H. W. EASL international Liver Congress 2017 (Hepatitis Debrief). https://livertree.easl.eu/easl/download/library/173802.2017. Accessed 01 Jul 2019

[6] Westhölter D, Hiller J, Denzer U, Polywka S, Ayuk F, Rybczynski M (2018). HEV-positive blood donations represent a relevant infection risk for immunosuppressed recipients. J. Hepatol..

[7] Horvatits T, Ozga AK, Westhölter D, Hartl J, Manthey CF, Lütgehetmann M (2018). Hepatitis E seroprevalence in the Americas: A systematic review and meta-analysis. Liver Int..

[8] Sarmiento-Silva RE, Arenas-Huertero F (2019). Hepatitis E in Latin America. Ann. Hepatol..

[9] Tengan FM, Figueiredo GM, Nunes AKS, Manchiero C, Dantas BP, Magri MC (2019). Seroprevalence of hepatitis E in adults in Brazil: A systematic review and meta-analysis. Infect. Dis. Poverty.

[10] Passos-Castilho AM, Reinaldo MR, Sena A, Granato CFH (2017). High prevalence of hepatitis E virus antibodies in Sao Paulo, Southeastern Brazil: Analysis of a group of blood donors representative of the general population. Braz. J. Infect. Dis..

[11] Passos-Castilho AM, de Sena A, Geraldo A, Spada C, Granato CF (2016). High prevalence of hepatitis E virus antibodies among blood donors in Southern Brazil. J. Med. Virol..

[12] Passos-Castilho AM, de Sena A, Reinaldo MR, Granato CF (2015). Hepatitis E virus infection in Brazil: Results of laboratory-based surveillance from 1998 to 2013. Rev. Soc. Bras. Med. Trop..

[13] Hering T, Passos AM, Perez RM, Bilar J, Fragano D, Granato C (2014). Past and current hepatitis E virus infection in renal transplant patients. J. Med. Virol..

[14] Zitelli, P., *et al.* The impact os HEV infection on the disease severity of patients with chronic hepatitis C. In *Annual Meeting of the American-Association-for-the Study-of-Liver-Diseases (AASLD)*. San Francisco, HEPATOLOGY, 935A-A (2018).

[15] Gomes-Gouvêa, M. S., *et al.* Evidence of hepatitis E virus infection in transplat liver recipients from Brazil. In *The Liver Meeting 2013: American Association for the Study of Liver Diseases (AASLD): HEPATOLOGY*, 1052A (2013).

[16] Kodani M, Kamili NA, Tejada-Strop A, Poe A, Denniston MM, Drobeniuc J (2017). Variability in the performance characteristics of IgG anti-HEV assays and its impact on reliability of seroprevalence rates of hepatitis E. J. Med. Virol..

[17] Abravanel F, Chapuy-Regaud S, Lhomme S, Miedougé M, Peron JM, Alric L (2013). Performance of anti-HEV assays for diagnosing acute hepatitis E in immunocompromised patients. J. Clin. Virol..

[18] Vollmer T, Diekmann J, Eberhardt M, Knabbe C, Dreier J (2016). Monitoring of anti-hepatitis E virus antibody seroconversion in asymptomatically infected blood donors: Systematic comparison of nine commercial Anti-HEV IgM and IgG assays. Viruses.

[19] Bricks G, Senise JF, Pott Junior H, Grandi G, Passarini A, Caldeira DB (2018). Seroprevalence of hepatitis E virus in chronic hepatitis C in Brazil. Braz. J. Infect. Dis..

[20] Bricks G, Senise JF, Pott-Jr H, Grandi G, Carnaúba-Jr D, de Moraes HAB (2019). Previous hepatitis E virus infection, cirrhosis and insulin resistance in patients with chronic hepatitis C. Braz. J. Infect. Dis..

[21] Castro VOL, Tejada-Strop A, Weis SMS, Stábile AC, de Oliveira SMVL, Teles SA (2019). Evidence of hepatitis E virus infections among persons who use crack cocaine from the Midwest region of Brazil. J. Med. Virol..

[22] Pandolfi R, de Almeida DR, Alves Pinto M, Kreutz LC, Frandoloso R (2017). In house ELISA based on recombinant ORF2 protein underline high prevalence of IgG anti-hepatitis E virus amongst blood donors in south Brazil. PLoS ONE.

[23] Hardtke S, Rocco R, Ogata J, Braga S, Barbosa M, Wranke A (2018). Risk factors and seroprevalence of hepatitis E evaluated in frozen-serum samples (2002–2003) of pregnant women compared with female blood donors in a Southern region of Brazil. J. Med. Virol..

[24] de Souza AJ, Gomes-Gouvêa MS, Soares MC, Pinho JR, Malheiros AP, Carneiro LA (2012). HEV infection in swine from Eastern Brazilian Amazon: Evidence of co-infection by different subtypes. Comp. Immunol. Microbiol. Infect. Dis..

[25] Gardinali NR, Barry AF, da Silva PF, de Souza C, Alfieri AF, Alfieri AA (2012). Molecular detection and characterization of hepatitis E virus in naturally infected pigs from Brazilian herds. Res. Vet. Sci..

[26] Oliveira-Filho EF, Dos Santos DR, Durães-Carvalho R, da Silva A, de Lima GB, Batista Filho AFB (2019). Evolutionary study of potentially zoonotic hepatitis E virus genotype 3 from swine in Northeast Brazil. Mem. Inst. Oswaldo Cruz..

[27] da Costa Lana MV, Gardinali NR, da Cruz RA, Lopes LL, Silva GS, Caramori Júnior JG (2014). Evaluation of hepatitis E virus infection between different production systems of pigs in Brazil. Trop. Anim. Health Prod..

[28] Capai L, Falchi A, Charrel R (2019). Meta-analysis of human IgG anti-HEV seroprevalence in industrialized countries and a review of literature. Viruses.

[29] Boutrouille A, Bakkali-Kassimi L, Crucière C, Pavio N (2007). Prevalence of anti-hepatitis E virus antibodies in French blood donors. J. Clin. Microbiol..

[30] Mansuy JM, Bendall R, Legrand-Abravanel F, Sauné K, Miédouge M, Ellis V (2011). Hepatitis E virus antibodies in blood donors, France. Emerg. Infect. Dis..

[31] Harritshøj LH, Kirkegaard-Klitbo DM, Mejer N, Panum I, Midgley SE, Ullum H (2019). Prevalence of anti-hepatitis E virus immunoglobulin G in HIV-infected individuals over three decades. Int. J. Infect. Dis..

[32] Ferreira AC, Gomes-Gouvêa MS, Lisboa-Neto G, Mendes-Correa MCJ, Picone CM, Salles NA (2018). Serological and molecular markers of hepatitis E virus infection in HIV-infected patients in Brazil. Arch. Virol..

[33] La Rosa G, Fratini M, Muscillo M, Iaconelli M, Taffon S, Equestre M (2014). Molecular characterisation of human hepatitis E virus from Italy: Comparative analysis of five reverse transcription-PCR assays. Virol. J..

[34] Leblanc D, Ward P, Gagné MJ, Poitras E, Müller P, Trottier YL (2007). Presence of hepatitis E virus in a naturally infected swine herd from nursery to slaughter. Int. J. Food Microbiol..

